# Meta-Analysis Reveals a Bilingual Advantage That Is Dependent on Task and Age

**DOI:** 10.3389/fpsyg.2020.01458

**Published:** 2020-07-24

**Authors:** Anna T. Ware, Melissa Kirkovski, Jarrad A. G. Lum

**Affiliations:** Cognitive Neuroscience Unit, School of Psychology, Deakin University, Geelong, VIC, Australia

**Keywords:** bilingual advantage, meta-analysis, executive functioning, publication bias, bilingualism

## Abstract

Debate continues on whether a bilingual advantage exists with respect to executive functioning. This report synthesized the results of 170 studies to test whether the bilingual advantage is dependent on the task used to assess executive functioning and the age of the participants. The results of the meta-analyses indicated that the bilingual advantage was both task- and age-specific. Bilinguals were significantly faster than monolinguals (Hedges' *g* values ranged from 0.23 to 0.34), and significantly more accurate than monolinguals (Hedges' *g* values ranged between 0.18 and 0.49) on four out of seven tasks. Also, an effect of age was found whereby the bilingual advantage was larger for studies comprising samples aged 50-years and over (Hedges' *g* = 0.49), compared to those undertaken with participants aged between 18 and 29 years (Hedges' *g* = 0.12). The extent to which the bilingual advantage might be due to publication bias was assessed using multiple methods. These were Egger's Test of Asymmetry, Duval and Tweedie's Trim and Fill, Classic Fail-Safe *N*, and PET-PEESE. Publication bias was only found when using Egger's Test of Asymmetry and PET-PEESE method, but not when using the other methods. This review indicates that if bilingualism does enhance executive functioning, the effects are modulated by task and age. This may arise because using multiple languages has a highly specific effect on executive functioning which is only observable in older, relative to younger, adults. The finding that publication bias was not uniformly detected across the different methods raises questions about the impact that unpublished (or undetected) studies have on meta-analyses of this literature.

## Introduction

An issue of ongoing debate concerns whether bilingualism enhances cognitive functions (Bialystok and Martin, 2004; Bialystok, 2011; Paap and Greenberg, 2013; Paap and Sawi, 2014; Pelham and Abrams, 2014), particularly, executive functioning (Bialystok, 1999, 2011; Bialystok et al., 2005; Poulin-Dubois et al., 2011; Marzecová et al., 2013a,b; Pelham and Abrams, 2014; Antón et al., 2016). This has been examined in over one-hundred studies (Lehtonen et al., 2018; Paap, 2019) and has been the subject of several narrative (Hilchey and Klein, 2011; Hilchey et al., 2015; Paap et al., 2015) and quantitative literature reviews (Adesope et al., 2010; Lehtonen et al., 2018; Donnelly et al., 2019; Paap, 2019). In this report, meta-analysis was used to examine three outstanding issues. The first was whether the task used to assess executive functioning might influence the extent to which the bilingual advantage is observed. The second was whether the magnitude of the bilingual advantage changes across the lifespan. The third was whether the bilingual advantage might be attributed to publication bias (Lehtonen et al., 2018; Donnelly et al., 2019; Paap, 2019).

It has been proposed that learning and using multiple languages enhances executive functioning (Bialystok, 1999, 2010, 2011; Bialystok and Martin, 2004; Carlson and Meltzoff, 2008; Bialystok and Viswanathan, 2009; Bialystok et al., 2010; Prior and MacWhinney, 2010). According to one view, in order to use multiple languages, one needs to inhibit the non-target language and then select the target language (Marian and Spivey, 2003; Marian et al., 2003; Green and Abutalebi, 2013). This constant switching and selection of different languages has been proposed to enhance executive functioning, even for non-linguistic tasks (e.g., Green, 1998; Bialystok, 1999, 2010, 2011).

### Past Reviews Examining the “Bilingual Advantage” for Executive Functioning

A large number of studies have been published examining whether a bilingual advantage exists for performance on executive functioning tasks (e.g., Hernandez et al., 2010; Antón et al., 2014; von Bastian et al., 2016). Furthermore, this literature has since been the subject of several reviews (Adesope et al., 2010; Hilchey and Klein, 2011; Hilchey et al., 2015; Lehtonen et al., 2018; Donnelly et al., 2019; Paap, 2019). Interestingly, these reviews have not reached the same conclusion.

Hilchey and Klein's (2011) systematic review initially found evidence for a bilingual advantage. This review summarized the results of 13 studies comprising adult and pediatric samples on three executive functioning tasks. These were the Simon, Flanker, and Attentional Network Tasks. A synthesis of individual study results revealed bilinguals had faster reaction times on these tasks. However, across the studies, the bilinguals did not consistently show superior performance on the components of these tasks that assess executive functioning. Thus, these findings suggest that bilingualism may improve general processing speeds rather than executive functioning.

In another review, evidence was found that bilingualism is associated with superior executive functioning. Adesope et al. (2010) used meta-analysis to examine whether there were differences between bilinguals and monolinguals with respect to meta-linguistic awareness, meta-cognitive awareness, working memory, abstract and symbolic reasoning, attentional control and problem solving. A total of 63 studies were included in this review. Relevant to the current report were analyses examining attentional control, as this construct aligns closely with executive functioning. Results of the meta-analysis revealed a bilingual advantage with respect to metalinguistic awareness (Hedges' *g* = 0.33, *p* < 0.01), working memory (Hedges' *g* = 0.48, *p* < 0.01), and abstract and symbolic reasoning (Hedges' *g* = 0.57, *p* < 0.01). However, the largest difference favoring bilinguals, was observed on the meta-analyses examining attentional control (Hedges' *g* = 0.96, *p* < 0.01). This pattern of results suggests a bilingual advantage that is most pronounced in the area of executive functioning.

Adesope et al. (2010) also examined whether the magnitude of the bilingual advantage was influenced by publication bias. Publication bias describes the occurrence whereby studies with positive or statistically significant findings are more likely to be published than those with non-significant findings (Easterbrook et al., 1991). In meta-analysis, this can lead to an overestimation of the “true” or “population” effect size, since only studies with significant findings are identified and included in the analysis. This issue has been raised as a concern in the bilingual advantage literature (de Bruin et al., 2015b). Adesope et al. examined this issue by assessing the number of un-retrieved or unidentified studies required to overturn their significant results. This was achieved by computing the Classic Fail-Safe *N* and Orwin's Fail-Safe *N* and then, comparing these values to the 5*k* + 10 guideline, where *k* denotes the number of effect sizes or studies included in the meta-analysis (Rosenthal, 1979). Using this approach, both Fail-Safe *N* values exceeded the 5*k* + 10 critical value. It was therefore suggested that publication bias was unlikely to be influencing the meta-analysis findings.

A systematic review by van den Noort et al. (2019) also reported a bilingual advantage in executive functioning. This review examined the results of 46 studies comparing bilingual and monolingual children and adults, on tasks assessing executive functioning. To synthesize the results from this literature, the authors summed the number of studies that reported a significant bilingual advantage. Using this approach, 54.3% of the studies reported that a bilingual advantage was present. It was also noted that 56.4% of studies reporting a significant bilingual advantage comprised adult samples, whereas 42.8% comprised pediatric samples. Differences in brain structure between adults and children were proposed to explain this trend in the literature. The suggestion was that in children, the regions of the brain that support executive functioning are still developing. As such, the effect of bilingualism on executive functioning may also be more variable during childhood compared to adults. Publication bias was not assessed in this review.

Reviews have also emerged questioning the magnitude and existence of the bilingual advantage. Donnelly et al. (2019) undertook a meta-analysis summarizing the results of 80 studies that compared executive functioning in bilingual and monolingual individuals. Initially a significant bilingual advantage was found, however, the observed effect size was small (Hedges' *g* = 0.11, *p* = 0.007). Publication bias was first assessed using Egger's Test of Asymmetry (Egger et al., 1997). Using this approach, publication bias was not found to be present. However, when the effect size was corrected for publication bias using the PET-PEESE model (Stanley and Doucouliagos, 2014), the bilingual advantage was no longer found to be significant.

Donnelly et al. (2019) also undertook a series of moderator analyses to examine potential variables influencing study level effect sizes. One moderator analysis examined whether average effect sizes differed between studies undertaken with children (participants aged 13-years or younger), younger adults (participants aged between of 15–40 years), and older adults (participants aged 60-years or older). However, no effect of age on effect sizes was found. Another moderator analysis examined whether effect sizes differed between the tasks used to examine executive functioning in the included studies (i.e., Flanker, Simon, Stroop, and Attentional Network Tasks). Again, for this analysis, task type was not found to moderate effect sizes.

Similar findings were reported by Lehtonen et al. (2018) who undertook a meta-analysis summarizing the results of 152 published and unpublished studies comparing bilingual and monolingual adults on executive functioning tasks. Initially the meta-analysis revealed a significant bilingual advantage, but the effect size was small in magnitude (Hedges' *g* = 0.06, *p* < 0.05). However, after correcting for publication bias using the PET-PEESE model (Stanley and Doucouliagos, 2014), the difference between bilinguals and monolinguals was no longer significant. (Hedges' *g* = −0.08, *p* = 0.099). Further analyses revealed that task and age were not significant moderators of study level effect sizes. The key finding from this review is that the bilingual advantage may be an artifact of publication bias.

Paap (2019) also presented evidence suggesting the bilingual advantage may be attributed to publication bias. This review summarized data from 109 studies. The included studies measured executive functioning using either the Stroop, Simon, Flanker, or Attentional Network Tasks. In one analysis examining differences in executive functioning, a bilingual advantage was initially found (Hedges' *g* = 0.11, *p* < 0.001). However, after correcting for publication bias using the PET-PEESE model, the average effect size approached zero and the difference between the two language groups was no longer significant (Hedges' *g* = −0.02, *p* = 0.708).

### The Current Meta-Analysis

Results from multiple meta-analyses are yet to determine the effect of bilingualism on executive functioning. In this review, 170 published and unpublished studies were identified to examine three outstanding issues. The first concerns whether the type of task used to measure executive functioning moderates the bilingual advantage effect. This may be expected given that the types of task used in this literature to measure this aspect of cognitive functioning are not highly correlated. For example, the correlation between the Simon and Flanker tasks has been found to be low (*r* = 0.14) and non-significant (Keye et al., 2009; Paap and Greenberg, 2013). Additionally, the correlations between the Flanker, Simon and Stroop tasks have been found to be less than 0.2 and non-significant (Stins et al., 2005). Based on these correlations it is possible to perform well on one task but not the other.

It is still unclear whether the bilingual advantage is task specific. This issue is important to address since in three previous meta-analyses, effect sizes from different tasks were combined in order to compute an overall average (Adesope et al., 2010; Hilchey and Klein, 2011; Hilchey et al., 2015). One potential problem with this approach is that it assumes different executive functioning tasks assess the same cognitive and/or neural processes. However, this may not be the case.

The second issue addressed in the current report concerns whether the age of the participants is moderating effect sizes. In one review (van den Noort et al., 2019), the bilingual advantage was found to be more commonly reported in adults compared to children. However, this result has not always been found (Lehtonen et al., 2018; Donnelly et al., 2019) and in two reviews (Adesope et al., 2010; Hilchey and Klein, 2011), this variable was not examined as a moderator. There is still inconsistency regarding the effects of age on the magnitude of the bilingual advantage.

A third issue concerns whether the bilingual advantage is an artifact of publication bias. In three meta-analyses (Lehtonen et al., 2018; Donnelly et al., 2019; Paap, 2019), evidence was presented suggesting this was the case. However, not all meta-analyses have found evidence to support this claim (Adesope et al., 2010). It is interesting to note that the meta-analyses which found evidence of publication bias used the PET-PEESE model. Meta-analyses that did not find evidence of publication bias used the Fail-Safe *N*, Duval and Tweedie's Trim and Fill method or Egger's Test of Asymmetry.

Quantifying and correcting the impact of publication bias in meta-analysis is problematic. This is because there is no universally accepted method that achieves this goal (Peters et al., 2007; Idris and Ruzni, 2012; Gervais, 2015). One noted problem with correcting for publication bias using PET-PEESE is that this approach may overestimate the effect of publication bias (Gervais, 2015). The opposite appears to be the case for other methods such as the Fail-Safe *N*, Duval and Tweedie's Trim and Fill or Egger's Test of Asymmetry (Peters et al., 2007). A stronger case for the argument that the bilingual advantage solely reflects publication bias could be made if this type of bias was present using different assessment methods.

### Objectives of the Current Report

This report examined whether a bilingual advantage exists with respect to executive functioning. Data from 170 studies comparing monolingual and bilingual individuals on commonly used tests of executive functioning were summarized using meta-analysis. The primary objectives of this report were to examine whether (i) the type of task used to assess executive functioning influenced the effect size measuring differences between bilinguals and monolinguals, (ii) whether the age of participants influenced effect sizes and, (iii) whether detecting publication bias depends on the type of method used in this literature.

## Methods

### Study Design

This report included both published and unpublished studies. Published studies were identified by searching the following electronic databases: Psychological Information Database (PsycINFO), Ageline, Medical Literature Analysis and Retrieval System Online (MEDLINE), Cumulative Index to Nursing and Allied Health Literature (CINAHL), Education Resources Information Center (ERIC), and Excerpta Medical Database (EMBASE). The search strategy used to identify relevant studies involved searching titles and abstracts for records containing synonyms for “bilingualism” and “executive functioning.” The complete search syntax used to search each database is available for download via the Open Science Framework (https://osf.io/ykp5w/?view_only=1d90f8c86146466e8dcfb6c4d23a334f).

The first search was executed in January, 2017. An additional search was undertaken in March, 2018 and then again in December, 2019 to ensure that any records published since the initial search were also included in the meta-analysis. Unpublished studies were found by searching the reference lists from three recent reviews (Lehtonen et al., 2018; Donnelly et al., 2019; Paap, 2019). This method was chosen to identify unpublished literature as there is no consensus about which databases used to search for gray literature. In this review we also wanted to ensure that the analyses of unpublished literature would include the same studies used in past meta-analysis, which was achieved using this method.

### Study Inclusion and Exclusion Criteria

Studies were included in this review if they met the following criteria. First, participants in each study needed to be healthy adults or children. Studies undertaken with clinical samples (e.g., ADHD or ASD) were excluded. In cases where a study presented data from both clinical and healthy samples (e.g., Mor et al., 2015), only data from healthy participants were extracted. Second, the study needed to compare a group comprising bilingual participants to a group comprising monolingual participants on an executive functioning task. Studies presenting data from only bidialectal (Antoniou et al., 2016; Wu et al., 2016; Scaltritti et al., 2017) or trilingual groups were excluded. Where a study included both bilingual and trilingual/bidialectal samples, only data from bilingual participants were extracted. This criterion aimed to find studies with fairly homogenous participants with respect to language functioning.

Third, the study needed to have measured executive functioning using either the Stroop Task, Simon Task, Attentional Network Task, Flanker Task, Trail Making Test, Task-Switching Paradigms, and/or Card Sort Tasks. These tasks were identified through a process using studies from the first search. The tasks used in each of these studies were noted. After examination, the authors decided that any task used in less than seven publications was to be excluded. The goal here was to reduce variability between study findings due to methodological differences. Also, the tasks included in this meta-analysis have been found to be valid measures of executive functioning (Eriksen and Eriksen, 1974; MacLeod, 1991; Rubinstein et al., 2001; Zelazo, 2006; Hommel, 2011; Salthouse, 2011). In this meta-analysis, average effect sizes (described below) were computed separately for each of these tasks. Studies that used non-standard versions of the aforementioned tasks (Coderre et al., 2013; Calvo and Bialystok, 2014) were excluded. Details on task exclusion criteria and an overview of each task are available for download via the Open Science Framework (https://osf.io/ykp5w/?view_only=1d90f8c86146466e8dcfb6c4d23a334f).

### Study Selection

**Figure 1:**
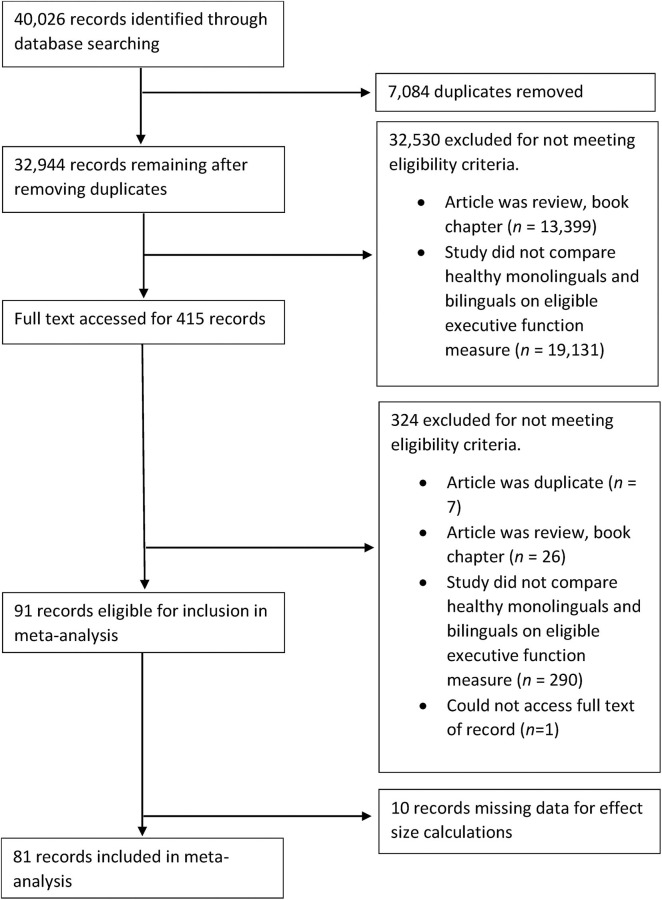
PRISMA flowchart detailing the first search of the literature undertaken in January, 2017.

**Figure 2:**
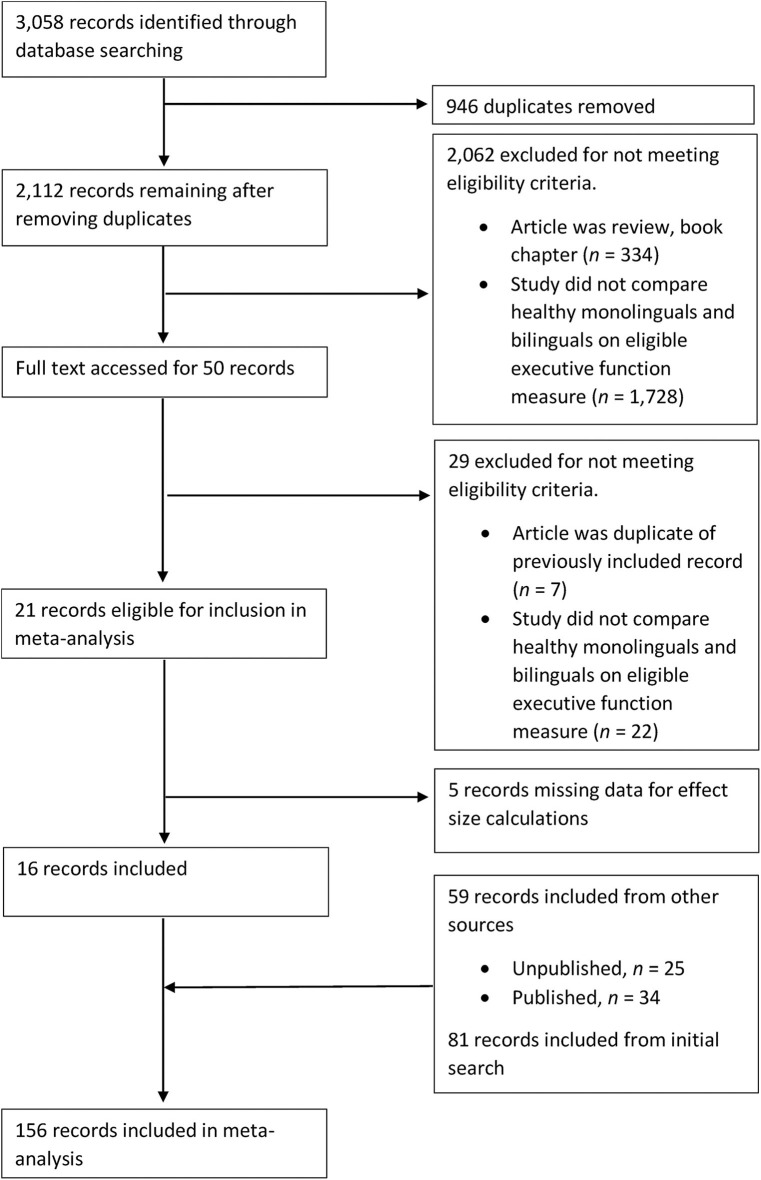
PRISMA flowchart detailing the second search of the literature undertaken in March, 2018.

**Figure 3:**
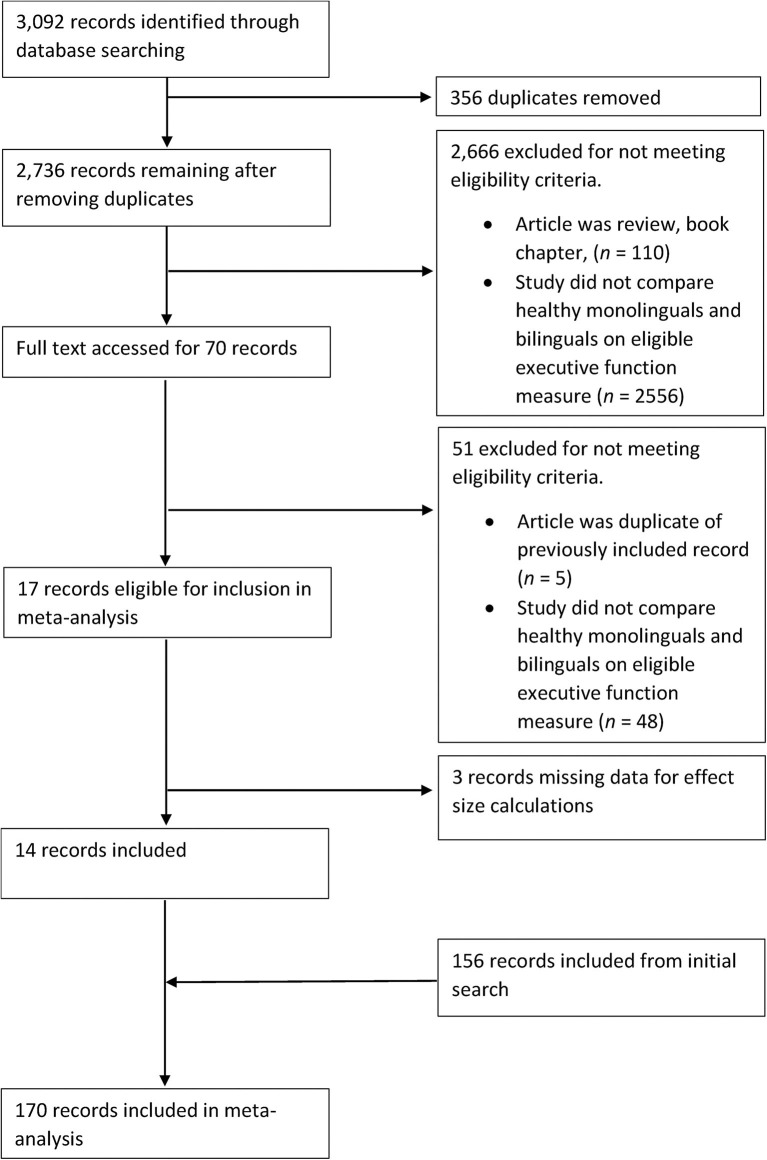
PRISMA flowchart detailing the third search of the literature undertaken in December, 2019.

The first search (completed in January, 2017) led to the identification of 81 studies that met the aforementioned criteria. The second search (completed in March, 2018) led to the identification of an additional 16 studies, and the third search (completed in December, 2019), an additional 14 studies. Finally, 59 unpublished studies were identified from the reference lists of three recent relevant reviews (Lehtonen et al., 2018; Donnelly et al., 2019; Paap, 2019). A total of 170 studies were included in the meta-analyses. Figures 1–3 present PRISMA flow charts detailing the screening process of articles obtained from the first, second and third round of searches respectively.

### Effect Size Calculations and Data Extraction Procedures

Data was extracted from each study so that Hedges' *g* (Hedges and Olkin, 1985) could be computed. Hedges' *g* measures differences between groups in standard deviation units, adjusted for sample size. For each study, data were extracted in order to compute effect sizes for the Stroop Task, Simon Task, Attentional Network Task, Flanker Task, Trail Making Test, Task-Switching Paradigms, and Card Sort Tasks. It was common for studies to quantify performance using accuracy, reaction time, and/or a measure of an interference effect. Effect sizes were computed separately for each of these variables.

For each task, data were extracted so that separate effect sizes could be obtained for incongruent and congruent trials, as well as for an interference effect. Congruent trials place the least demands on executive functioning processes. Performance on these trials has been interpreted to reflect general processing speed (Bialystok et al., 2005; Hilchey and Klein, 2011). Incongruent trials place greater demands on executive functioning processes, as one is required to inhibit a response in favor of another (Miyake et al., 2000; Nigg, 2000). For example, on the Stroop task, participants are asked to state the color of the font of a presented word. Congruent trials are those where the word and the color in which the word is presented, are the same; the word “GREEN” presented in green ink. Conversely, incongruent trials involve a mismatch between the word, and its color. For example, the word “GREEN” presented in red ink. In this literature, an additional measure used to examine executive functioning processes involves computing the difference in response times between congruent and incongruent trials. This variable measures the increased cognitive demands required to resolve incongruent, relative to congruent, trials. On this variable, smaller values indicate that task is placing fewer demands on executive functioning processes. This variable is referred to as the interference effect in this report.

Since the focus of this report was to examine the effects of bilingualism on executive functioning, effect sizes from incongruent trials and interference scores were primarily of interest. However, for completeness, data were also extracted to compute effect sizes summarizing performance on congruent trials as well. For congruent trials, incongruent trials, and the interference effect, positive Hedges' *g* values indicate superior performance by the bilingual group. Effect sizes for these variables were computed separately for accuracy and reaction time data.

Each study effect size was also coded based on the age of the sample from the study. Effect sizes extracted from studies where the mean age of the sample was less than 18-years, were coded as representing a “Child” sample. We did not examine child and adolescent samples separately. This was because only one study was identified in our search, that was undertaken with an adolescent sample. All other pediatric samples were undertaken with participants who were under the age of 13-years. Data extracted from experiments where the mean sample age was between 18 and 29 were coded as “Young Adult.” “Adult” samples comprised effect sizes from experiments where the mean age of the sample was between 30 and 49 years of age. Finally, effect sizes extracted from studies where participants were 50-years of age or older were coded as “Older Adults.” Study level effect sizes used in the meta-analyses are available for download via the Open Science Framework (https://osf.io/ykp5w/?view_only=1d90f8c86146466e8dcfb6c4d23a334f).

### Meta-Analytic Procedures

#### Averaging Effect Sizes

Meta-analyses were undertaken to examine whether (i) the difference between bilingual and monolingual groups varied between executive functioning tasks, and, (ii) if the average effect size between bilingual and monolingual groups varied in magnitude by age group.

To examine the effect of task on the bilingual advantage, the average effect size was computed separately for each of the studied tasks. That is, meta-analyses were conducted separately for the Stroop Task, Simon Task, Attentional Network Task, Flanker Task, Trail Making Test, Task-Switching Paradigms, and Card Sort Tasks. For all meta-analyses, study level effect sizes were averaged using a random effects model (Hedges and Olkin, 1985). Effect sizes were averaged separately for incongruent and congruent trials and for accuracy, reaction time, and the interference effect variable.

Two approaches were used to examine whether the bilingual advantage differed between tasks. First, for each task and variable (i.e., congruent trials, incongruent trials, interference effect), we examined if the average effect size was significant. For these and all analyses, alpha was set at 0.05. Second, mixed-effects subgroup analyses (Borenstein et al., 2011) tested whether effect sizes for each variable was significantly different between the tasks. A similar approach was used to examine whether effect sizes varied across the different age bands.

### Publication Bias

Multiple methods were used to detect publication bias in this report. Specifically, publication bias was assessed using Egger's Test of Asymmetry, Duval and Tweedie's Trim and Fill method, Classic Fail-Safe *N*, and PET-PEESE. The rationale for using a wide range of bias tests was to see whether findings of publication bias were specific to one test, or present across multiple tests. This was important to determine since no consensus has been reached regarding the most accurate method to detect publication bias (Peters et al., 2007; Idris and Ruzni, 2012; Gervais, 2015). Additionally, we empirically quantified the effect of publication bias by computing the average effect size for all variables separately, for published and unpublished studies. Mixed-effects analyses were undertaken to compare whether the average effect sizes from published and unpublished studies were significantly different. This approach served as a benchmark from which to compare the results from the aforementioned methods for assessing publication bias which are based on a theoretical distribution of effect sizes.

#### Egger's Test of Asymmetry

Egger's Test of Asymmetry assesses for publication bias using the properties of funnel plots. Funnel plots show the association between study level effect sizes and standard error (Egger et al., 1997). When publication bias is absent, effect sizes are symmetrically distributed around the average of the effect sizes. When publication bias is present, the funnel plot has an asymmetrical shape. This asymmetry is formally tested using Egger's Test of Asymmetry. When this test returns a significant result, publication bias may be present.

#### Duval and Tweedie's Trim and Fill

Duval and Tweedie's (2000) Trim and Fill method uses the properties of the funnel plot to correct for publication bias. In this method, missing effect sizes that give rise to an asymmetrical funnel plot are imputed. In the current report, missing studies were assumed to have negative effect size values. That is, missing effect sizes were assumed to show that monolinguals performed better than bilinguals on a given variable. An imputed average effect size can then be compared with the observed average effect size. A limitation of this approach is that it often underestimates the extent of bias and can be too lenient in the correction (Idris and Ruzni, 2012).

#### Classic Fail-Safe N

The Classic Fail-Safe *N* estimates the number of hypothetical un-retrieved studies required to return a significant average effect size to non-significance. When the Fail-Safe *N* is low, publication bias is possible. This is because the observed result in a meta-analysis could be overturned by a small number of unpublished or un-retrieved studies that were missed during the search process. When the Fail-Safe *N* value is high, publication bias is unlikely. Rosenthal (1979) suggested that when the Fail-Safe *N* value is greater than 5*k* + 10, where *k* is the number of studies (or effect sizes) included in the meta-analysis, publication bias is unlikely. This approach was used in the current report.

#### PET-PEESE

The PET-PEESE method (Stanley and Doucouliagos, 2014) is a relatively more recent approach for assessing publication bias. This method uses weighted least squares regression to predict individual study effect sizes from their standard error. In this model, the intercept is considered to represent the average effect size after controlling for potential publication bias. One criticism of the PET-PEESE model is that it overestimates the effect of publication bias on the average effect size (Gervais, 2015).

## Results

Results from the meta-analyses are presented in three sections. The first section presents analyses examining whether the bilingual advantage is more likely to be observed on some executive functioning tasks over others. The second section presents analyses examining whether effect sizes vary significantly between child, young adult, adult and older adult samples. The third section presents results from the assessment of publication bias.

### Is the Bilingual Advantage Task Dependent?

**Figure 4:**
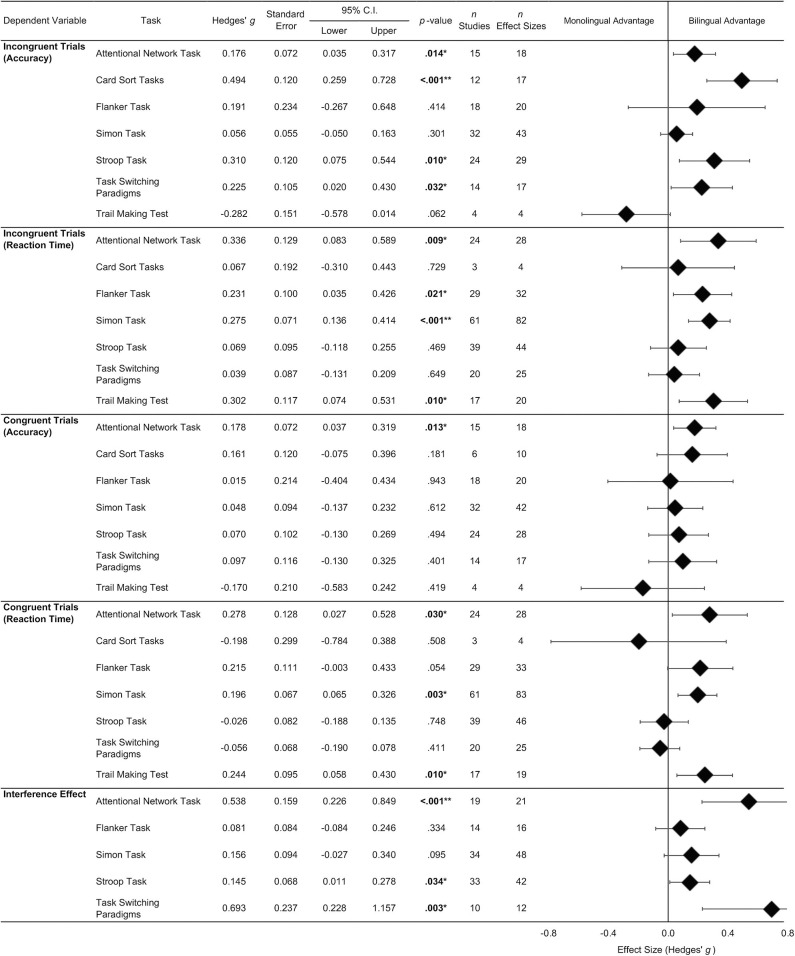
Average effect sizes reported by Task, Trial Type, and for accuracy and reaction time data. No interference effect/cost effect sizes were available for the Card Sort Tasks and Trail Making Test. ^*^p < 0.05; ^**^p < 0.001.

Results from the meta-analyses examining differences between bilingual and monolingual participants for the studied executive functioning tasks are presented in Figure 4. This figure shows the averaged effect sizes reported by task.

Results from the meta-analyses summarizing performance from the different tasks on the incongruent trials are considered first. With respect to accuracy data, averaged effect sizes ranged from −0.282 to 0.485. Effect sizes for four out of the seven meta-analyses were significant. These were from the Attentional Network Task, Stroop Task, Task-Switching Paradigms, and Card Sort Tasks. For these tasks, the effect sizes ranged from small to medium and were positive. This indicates that bilinguals were more accurate than monolinguals on incongruent trials for these tasks. Average effect sizes for the other tasks were non-significant.

Mixed-effects subgroup analysis revealed that the effect sizes for accuracy on incongruent trials varied significantly by task (*p* < 0.001). This result indicates that the type of task used to assess executive functioning influenced the magnitude of the difference between bilinguals and monolinguals. To explore this result further, mixed-effects subgroup analyses were conducted to examine differences in pairs of effect sizes. Results from these comparisons are summarized in Table 1.

**Table 1:** *p*-values from subgroup analyses comparing pairs of accuracy effect sizes from incongruent trials.

**Task**	**1. Stroop Task**	**2. Simon Task**	**3. Attentional Network Task**	**4. Flanker Task**	**5. Task Switching Paradigms**	**6. Trail Making Test**	**7. Card Sort Tasks**
1. Stroop Task	–						
2. Simon Task	0.054	–					
3. Attentional Network Task	0.338	0.185	–				
4. Flanker Task	0.651	0.574	0.951	–			
5. Task Switching Paradigms	0.594	0.154	0.700	0.895	–		
6. Trail Making Test	0.002^*^	0.035^*^	0.006^*^	0.089	0.006^*^	–	
7. Card Sort Tasks	0.288	0.001^*^	0.021^*^	0.258	0.092	<0.001^*^	–

* *p < 0.05*.

Table 1 shows that the average effect size for Card Sort Tasks was significantly larger than the effect sizes for the Attentional Network Task, Simon Task and Trail Making Test. Effect sizes from the Stroop Task, Simon Task, Attentional Network Task, and Task-Switching paradigms were significantly larger than the effect size for the Trail Making Test. Results from all other comparisons were not statistically significant.

Effect sizes from reaction times on incongruent trials ranged from 0.039 to 0.336. On four tasks, the Attentional Network Task, Simon Task, Flanker Task, and the Trail Making Test, the average effect size was positive and significant, indicating a bilingual advantage. On these tasks, the average finding in the literature is that bilinguals respond faster than monolinguals on incongruent trials. All other analyses were not significant.

Mixed-effects subgroup analysis comparing differences in reaction time effect sizes from incongruent trials was not significant (*p* = 0.183). This result indicates that the task used did not influence the magnitude of the difference between the groups on this variable.

Analyses examining the influence of executive functioning tasks on effect sizes from congruent trials are now considered. For accuracy data, effect sizes ranged from −0.170 to 0.178. On the Attentional Network Task, the average effect size was positive and significant, although small in magnitude. This result indicates that for congruent trials on this task, bilinguals are more accurate than monolinguals. There were no other significant results from this set of analyses.

Mixed-effects subgroup analyses comparing differences in these task-level average effect sizes were not significant (*p* = 0.727). Thus, for accuracy data obtained from congruent trials, the type of task used was not influencing effect sizes.

Effect sizes for reaction time data collected from congruent trials ranged from −0.198 to 0.278. The average effect sizes from the Trail Making Test, Attentional Network Task, and Simon Task were all positive and significant, though small. This result indicates that for these tasks on congruent trials, the average finding is that bilinguals have faster reaction times compared to monolinguals. All other comparisons were non-significant.

Mixed-effects subgroup analyses indicated that task influenced the magnitude of the difference between bilinguals and monolinguals (*p* = 0.014) with respect to reaction time on congruent trials. Results from pairwise comparisons are presented in Table 2. The average effect sizes from the Simon Task, Attentional Network Task, and Trail Making Test were significantly larger than the effect size from the Stroop Task. Additionally, effect sizes from the Flanker, Simon, Attentional Network, and Trail Making tasks were significantly larger than the effect size for Task-Switching Paradigms.

**Table 2:** *p*-values from subgroup analyses comparing pairs of reaction time effect sizes from congruent trials.

**Task**	**1. Stroop Task**	**2. Simon Task**	**3. Attentional Network Task**	**4. Flanker Task**	**5. Task Switching Paradigms**	**6. Trail Making Test**	**7. Card Sort Tasks**
1. Stroop Task	–						
2. Simon Task	0.036^*^	–					
3. Attentional Network Task	0.046^*^	0.57	–				
4. Flanker Task	0.081	0.883	0.710	–			
5. Task Switching Paradigms	0.778	0.008^*^	0.021^*^	0.037^*^	–		
6. Trail Making Test	0.031^*^	0.680	0.831	0.843	0.010^*^	–	
7. Card Sort Tasks	0.579	0.198	0.143	0.195	0.643	0.159	–

* *p < 0.05*.

Finally, analyses examining the influence of task type on effect sizes for interference effects are presented. The effect sizes for these meta-analyses ranged from 0.081 to 0.693. For three tasks, the Attentional Network Task, Stroop Task, and Task-Switching Paradigms, the average effect sizes were significant and positive. This suggests that on average, on these tasks, bilinguals display a smaller interference effect relative to monolinguals. The effect sizes for the Flanker and Simon tasks were small and non-significant. Mixed-effect subgroup analyses indicated that task influenced the magnitude of the difference between the groups (*p* = 0.022). Results from pairwise comparisons are presented in Table 3. The average effect sizes for the Attentional Network Task and Task-Switching Paradigms were significantly larger than the effect sizes for the Stroop, Simon, and Flanker tasks.

**Table 3:** *p*-values from subgroup analyses comparing pairs of effect sizes from the interference effect.

**Task**	**1. Stroop Task**	**2. Simon Task**	**3. Attentional Network Task**	**4. Flanker Task**	**5. Task Switching Paradigms**
1. Stroop Task	–				
2. Simon Task	0.924	–			
3. Attentional Network Task	0.023^*^	0.039^*^	–		
4. Flanker Task	0.554	0.552	0.011^*^	–	
5. Task Switching Paradigms	0.026^*^	0.035^*^	0.587	0.015^*^	–

* *p < 0.05*.

*The bilingual advantage across separate tasks: An interim Summary*. As noted earlier, positive effect sizes from incongruent trials and the interference effect indicate a bilingual advantage for executive functioning. The findings reported above indicate superior executive functioning in bilinguals that is consistently observed on the Attentional Network Task. A bilingual advantage was not consistently found on the other examined tasks. Subgroup analyses revealed that task type moderated effect sizes. Thus, the bilingual advantage does not appear to be present on all tasks.

### Is the Bilingual Advantage Dependent on Age?

**Figure 5:**
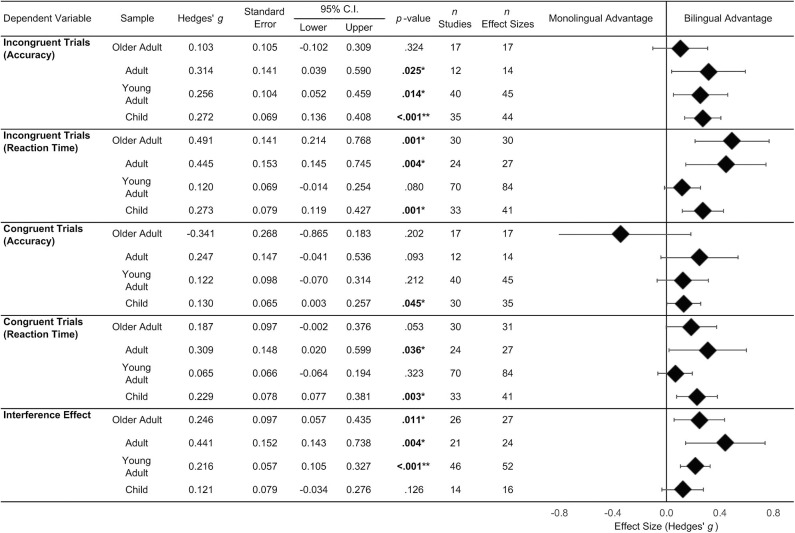
Average effect sizes reported by Age Group, Trial Type, and for accuracy and reaction time data. No interference effect, effect sizes were available for the Card Sort Tasks and Trail Making Test. ^*^p < 0.05; ^**^p < 0.001.

Figure 5 shows the results from the meta-analyses examining differences between bilinguals and monolinguals on congruent trials, incongruent trials, and the interference effect. Results are reported separately for each age group.

Meta-analyses conducted for accuracy on incongruent trials will be discussed first. On this variable the average effect size ranged from 0.103 to 0.314. The small effect sizes for adult, young adult and child samples were significant and positive. This suggests that for these age groups, bilinguals are more accurate than monolinguals on incongruent trials across the included tasks. Mixed-effect subgroup analysis revealed no significant difference in the averaged effect sizes between each age group (*p* = 0.529).

The effect sizes from reaction times for the different age bands on incongruent trials ranged from 0.120 to 0.491. The average effect sizes were significant for child, adult, and older adult samples. This suggests that on average, bilinguals in these age groups are faster than monolinguals on incongruent trials. Mixed-effect subgroup analysis revealed significant differences in the age-level effect sizes (*p* = 0.043). Results from pairwise comparisons are presented in Table 4. These analyses indicated that the average effect size from young adults was significantly smaller than the effect size from older adult samples.

**Table 4:** *p*-values from subgroup analyses comparing pairs of reaction time effect sizes from incongruent trials, by age.

**Age**	**1.Older adult**	**2. Adult**	**3. Young adult**	**4. Child**
1. Older adult	–			
2. Adult	0.825	–		
3. Young adult	0.018^*^	0.053	–	
4. Child	0.177	0.318	0.137	–

* *p < 0.05*.

The meta-analyses conducted for accuracy on congruent trials revealed effect sizes ranging from −0.341 to 0.247. A significant, small, effect size was observed for child samples. This indicates that the average finding for this group is that bilingual children are more accurate than monolingual children on congruent trials. Effect sizes for other age groups were small and non-significant. Mixed-effect subgroup analysis did not indicate significant differences in the effect sizes between the age groups (*p* = 0.293).

Average effect sizes for reaction time on congruent trials ranged from 0.187 to 0.309. Significant effect sizes were observed for adult and child samples, indicating that on average, bilinguals in these age groups were faster than monolinguals on congruent trials. There were no significant differences between bilinguals and monolinguals in the other two age samples. Mixed-effects subgroup analysis revealed no significant differences between the age group effect sizes (*p* = 0.268).

Last, the effect sizes for the interference effect ranged from 0.121 to 0.441. Positive, significant effect sizes were observed for the older adult, adult, and young adult samples suggesting that bilinguals in this age group have smaller interference effects than their monolingual peers. The effect size for child samples was comparatively smaller and non-significant. There were no significant differences between the age group level effect sizes (*p* = 0.293).

*The bilingual advantage across age groups: An interim Summary*. The main result to emerge from the above meta-analyses is that a significant bilingual advantage on variables assessing executive functioning was more likely to be observed in studies undertaken with older participants. In one analysis, age was moderating effect sizes. Typically, effect sizes from older adult samples were significantly larger than those from younger age groups.

### Evaluation of Publication Bias

Publication bias was separately examined for effect sizes computed from incongruent and congruent trials, both accuracy and reaction time, as well as for the interference effect. For each of these variables, effect sizes were averaged using a random effects model. These weighted average effect sizes are presented in Table 5 under “Observed Effect Size.” For these effect sizes, significant positive values were observed for four out of five variables, indicating a bilingual advantage.

**Table 5:** Results from Analyses of Publication Bias.

**Variable**	**Total number of effect sizes (*k*)**	**Published effect size (Hedges' *g*)**	**Unpublished effect size (Hedges' *g*)**	**Observed effect size (Hedges' *g*)**	**Imputed effect size**		**Eggers test of asymmetry (*p*-value)**	**Classic fail-Safe *N***	**Classic-fail-safe *N* critical value (computed using 5*k* + 10)**
					**Duval and tweedie's trim and fill method^a^ (Hedges' *g*)**	**PET-PEESE(Hedges' *g*)**			
Incongruent Trials									
Accuracy	118	0.273^**^	0.210^*^	0.261^**^	0.261^**^	−0.330^*^	<0.001^**^	2,939	600
Reaction Time	180	0.280^**^	−0.004	0.239^**^	0.239^**^	−0.442^*^	<0.001^**^	4,504	910
Congruent Trials									
Accuracy	109	0.117	0.093	0.108^b^	0.108	−0.002	0.865	–^b^	–^b^
Reaction Time	181	0.202^**^	−0.105	0.150^*^	0.150^*^	−0.334^*^	<0.001^**^	1,688	915
Interference Effect	116	0.335^**^	0.093	0.268^**^	0.268^**^	−0.328^*^	<0.001^**^	2,921	590

* *p < 0.05*;

** *p < 0.001*;

a *Missing studies were assumed to have negative effect sizes (i.e., monolingual evidencing superior performance than bilinguals)*.

b *Classic Fail Safe N cannot be computed for these tasks since observed average effect size was found to be non-significant*.

Also presented in Table 5 are the average effect sizes for each variable computed separately for published and unpublished studies. These effect sizes were averaged using a random effects model. Generally, effect sizes for published studies were larger compared to unpublished studies, but the magnitude of the difference varied between the variables. For example, the effect size from published studies computed using accuracy data from incongruent trials was 0.063 points larger than the corresponding effect size for unpublished studies. The effect size for the interference effect was 0.242 points larger for published studies compared to unpublished studies. Mixed-effects analysis was used to formally test whether there were significant differences between the effect sizes from published and unpublished studies. There was no significant difference between published and unpublished average effect sizes for accuracy on incongruent (*p* = 0.566) or congruent trials (*p* = 0.839). However, effect sizes from published studies were found to be significantly larger for reaction times on both incongruent (*p* = 0.013) and congruent trials (*p* = 0.007), as well as for the interference effect (*p* = 0.004). These analyses indicate that based on the studies included in this review, published studies had a larger effect size than unpublished studies for three out of the five variables.

Publication bias for each variable was assessed using the Classic Fail-Safe *N*, Egger's Test of Asymmetry, Duval and Tweedie's Trim and Fill method, and PET-PEESE. Results from each of these methods are presented in Table 5. The Fail-Safe *N* value indicated publication bias was unlikely. Rosenthal (1979) suggested that if the Fail-Safe *N* value was greater than 5*k* + 10, then it is unlikely that a significant meta-analysis result was due to publication bias. For all variables, the Fail-Safe *N* values were greater than the critical values estimated by the formula. However, Eggers Test of Asymmetry, indicated that publication bias was likely for four out of five of the variables.

The tests that both detect publication bias and estimate a corrected effect size, returned contradictory results. The imputed average effect size using Duval and Tweedie's Trim and Fill method indicated publication bias was unlikely. For all variables, the imputed average effect size was equal to the observed value. It should be noted this result was obtained by assuming missing studies all reported negative effect sizes (indicating a monolingual advantage). Conversely, results from PET-PEESE indicated publication bias was likely. This estimate for all variables was negative and significant, indicating a monolingual advantage. The results of the PET-PEESE analysis indicate the bilingual advantage is an artifact of publication bias.

## Discussion

This report used meta-analysis to examine the extent task and age influenced the bilingual advantage with respect to executive functioning. To our knowledge, this is the largest meta-analysis undertaken on this topic to date, in terms of the number of studies reviewed (*k* = 170). Three main findings emerged from this review. First, there is evidence to suggest that bilinguals may outperform monolinguals on some, but not all, executive functioning tasks. Second, a bilingual advantage in executive functioning was more likely to be present for studies undertaken with adult and older participants (i.e., aged over 30-years). Third, publication bias may be leading to an overestimation of effect sizes in meta-analyses examining the bilingual advantage, however, the extent of this overestimation is unclear.

### The Bilingual Advantage Appears to Be Task-Dependent

The results of the meta-analyses indicate that the bilingual advantage is moderated by the type of task used to assess executive functioning. Also, in this report, a bilingual advantage was consistently observed on the Attentional Network Task. This effect was found across all studied variables (i.e., congruent trials, incongruent trials, interference effect). For other tasks, a bilingual advantage was not consistently found on all variables. Based on this set of results, if bilingualism improves executive functioning, it appears to be most pronounced on the processes assessed by the Attentional Network Task.

It is interesting to note that while the bilingual advantage was present on the Attentional Network Task, the effect was generally absent for the Flanker Task. This result was surprising given the similarity between both tasks. Both require participants to respond to the direction of a center arrow and ignore two flanking arrows on either side. Also, both comprise congruent trials, where the flanking and center arrows are pointing in the same direction, and incongruent trials, where the flanking arrows are pointing in a different direction to the center arrow.

However, there is a methodological difference relating to the presentation of visual stimuli on the Attentional Network and Flanker Tasks which may provide new insights into the effects of bilingualism on cognitive functioning. On the Flanker Task, stimuli are typically presented in the center of the screen, following a fixation cross. However, on the Attentional Network Task, stimuli are presented either above or below the previous location of the central fixation cross. This particular feature of the Attentional Network Task is included as a measure of attention, as participants must orient their attention to the stimuli before responding (Fan et al., 2002). In this report, the meta-analyses indicated a bilingual advantage across all variables of the Attentional Network Task but not the Flanker Task. Thus, there may be a bilingual advantage which is specific to attention. That is, bilinguals are able to orient their attention to the stimuli faster than monolinguals. This may explain why the results of the meta-analyses indicated that, on average, studies reported that bilinguals outperformed monolinguals on congruent trials as well as incongruent trials and interference effects on the Attentional Network Task.

There are at least two explanations to account for our finding that the cognitive benefits of bilingualism vary across executive functioning tasks. One explanation is that bilingualism influences a specific neural network or set of cognitive processes that is not activated (or required) in all executive functioning tasks. Consequently, differences between bilingual and monolingual groups are only present on tasks that activate the neural networks or cognitive processes that are enhanced by bilingualism. For example, one mixed-effects subgroup analysis showed effect sizes observed for the Simon Task were significantly smaller than those from the Stroop Task. There is evidence to suggest that even though both tasks are valid measures of executive functioning, they elicit a different pattern of neural activation. Peterson et al. (2002) found that similar brain regions were involved in the Stroop and Simon tasks, however there was variation in the level of brain activation between the tasks. For example, both tasks led to increased activation in the inferior frontal, parietal, and dorsolateral prefrontal cortices and the anterior cingulate and supplementary motor area. This reflects that both tap into parts of the brain that support executive functioning (e.g., Curtis and D'Esposito, 2003; Kondo et al., 2004; Obeso et al., 2013). However, the Simon Task led to greater levels of activation in the superior and inferior regions of the parietal lobe. These regions of the brain support the allocation of attention (Behrmann et al., 2004). Thus, it could be that bilingualism has an effect that is region- or network-specific.

Secondly, the psychometric properties of executive functioning tasks might also explain the results of this meta-analysis. Differences in task reliability could lead to the same pattern of results observed in this report. This is because poor reliability attenuates effect sizes (Baugh, 2002; Paap, 2019). As the measurement error of a task increases (i.e., the reliability decreases), the effect size decreases. Differences in study level effect sizes may, in part, reflect differences in task reliability. For example, the Stroop Task may have had higher reliability than the Simon Task in the studies comparing bilingual and monolingual participants. In meta-analysis it is possible to correct average effect sizes for the impact of poor reliability (Baugh, 2002; Schmidt and Hunter, 2014). However, this correction requires knowing the reliability associated with the task being used in a study. In the studies reviewed for our meta-analyses, reliability of the executive functioning tasks was not reported. If reliabilities are reported in future research, the impact of measurement error on effect sizes can be estimated.

### The Bilingual Advantage Is Dependent on Age

A potentially novel finding in this report was that the bilingual advantage was consistently observed in samples aged between 30 and 49 years. For this age group, a bilingual advantage was observed on four variables (accuracy and reaction time on incongruent trials, reaction times on congruent trials and interference effect). Although the magnitude of the advantage was not significantly larger than in other age groups, this trend hints at a bilingual advantage that may be more consistent in studies undertaken with participants aged between 30 and 49 years. A second novel finding with respect to age was that the magnitude of the bilingual advantage for response times on incongruent trials was significantly larger in samples aged 50-years and above, compared to samples aged between 18 and 29 years of age. This suggests that the bilingual advantage may be larger in studies undertaken with older samples, compared to young adults.

Our finding that the age of participants influences effect sizes is not consistent with previous meta-analyses, in which age was not found to be a significant moderator of the bilingual advantage (e.g., Lehtonen et al., 2018; Donnelly et al., 2019). One explanation for this discrepancy between our review and others is the method used to correct for publication bias. Both Lehtonen et al. and Donnelly et al. used the PET-PEESE method to correct effect sizes before interpreting results. The PET-PEESE approach has been criticized for overestimating the effect of publication bias (Gervais, 2015). This issue is discussed in the next section (Publication Bias and the Bilingual Advantage) in detail. Using this method may have potentially masked age-related bilingual advantage differences.

If the bilingual advantage is greater in older samples, why might this be so? It has been documented that executive functioning often declines with age (Ardila and Rosselli, 1989; Parkin and Lawrence, 1994; Lustig et al., 2001; Bowles and Salthouse, 2003). One suggestion is that bilingualism reduces the effects of cognitive decline. That is, bilingualism may help protect against the decline of executive functions (Lee Salvatierra and Rosselli, 2011; Schroeder and Marian, 2012). One idea is that using multiple languages places constant demands on executive functioning which in turn, reduces the impact of age-related brain changes on this aspect of cognitive functioning (Fiszer, 2008). In older age groups, bilinguals may evidence superior executive functioning skills compared to monolinguals because this skill is relatively more preserved. In younger age groups, the bilingual advantage in executive functioning may be small or absent. This is because in younger age groups, executive functioning processes are already at their peak level of performance. Ceiling effects in executive functioning in younger age groups may lead to non-significant differences between bilinguals and monolinguals.

### Publication Bias and the Bilingual Advantage

A final issue addressed in this report is the extent to which the bilingual advantage can be considered an artifact of publication bias. The potential effect of publication bias on reviews examining the bilingual advantage was demonstrated by de Bruin et al. (2015a). In this study, authors examined conference abstracts from 1999 to 2012 and found that those reporting a bilingual advantage were more likely to be published. Meta-analyses have also suggested that the bilingual advantage may be an artifact of publication bias. Both Donnelly et al. (2019) and Paap (2019), initially reported a small bilingual advantage which became non-significant after using the PET-PEESE method to correct for publication bias.

The analyses of publication bias presented in this report questions whether the bilingual advantage can be completely attributed to publication bias. In this review, both the PET-PEESE method and Egger's Test of Asymmetry suggest that publication bias is present. A key issue emerging from our analyses is that it is unclear how much this publication bias is inflating the average effect sizes in meta-analyses examining the bilingual advantage. After adjusting the average effect size for publication bias using Duval and Tweedie's Trim and Fill method, a reliable bilingual advantage was still found. However, using the PET-PEESE approach the bilingual advantage was no longer present. An outstanding question to be addressed from our analyses is which approach most accurately corrects the observed effect size. This is an issue that will need to be addressed in future research evaluating meta-analysis methods.

However, in the context of the bilingual advantage, we tentatively suggest that the PET-PEESE method might be overestimating the effect of publication bias. In this report we computed average effect sizes separately for published and unpublished studies (See Table 5). It is noted that in all cases, the effect sizes for published studies were larger compared to unpublished. Across the studied variables, the difference in average effect sizes between published and unpublished studies ranged between 0.024 and 0.307. However, using the PET-PEESE method, the observed average effect size was reduced by between 0.110 and 0.681. This reduction is substantially larger than the difference we observed between published and unpublished studies. Therefore, we somewhat align our findings with the suggestion by Donnelly et al. (2019) that the magnitude of the advantage has been “overstated.” We do suggest, however, that it is not entirely clear whether publication bias completely explains the tendency for bilinguals to perform better on some cognitive tasks than monolinguals. Thus, research investigating how learning and using multiple languages affects cognitive functioning outside the linguistic domain is still needed.

### Limitations

In this report we were not able to examine other moderating variables that may also influence effect sizes. For instance, there are varying dimensions of bilingualism that are potential moderators of the bilingual advantage. These may include differences in language proficiency, age of acquisition and the context of language use (Green and Abutalebi, 2013). These variables were not examined systematically in this meta-analysis because this information was not available or was not reported with sufficient consistency to be analyzed. Consistent reporting of participant background characteristics will permit moderator analyses in future meta-analyses of this topic.

## Conclusion

The results of this report indicate that bilingualism can enhance cognitive functioning. However, the bilingual advantage is more likely to be observed on the Stroop, Simon, and Attentional Network tasks, and in participants over 50-years of age. Understanding why the advantage is most pronounced on these tasks and in this age group could advance our understanding of how learning and using multiple languages influences neural and cognitive functioning.

## Data Availability Statement

Publicly available datasets were analyzed in this study. This data can be found here: https://osf.io/ykp5w/?view_only=1d90f8c86146466e8dcfb6c4d23a334f.

## Author Contributions

JL and AW were responsible for the acquisition and analysis of data. MK, JL, and AW were all responsible for the interpretation of data and preparation of the manuscript.

## Conflict of Interest

The authors declare that the research was conducted in the absence of any commercial or financial relationships that could be construed as a potential conflict of interest.
